# Current Status and Future Prospects of Proton MR Spectroscopy of the Breast with a 1.5T MR Unit

**DOI:** 10.1155/2010/781621

**Published:** 2010-09-26

**Authors:** Mitsuhiro Tozaki, Katsuya Maruyama

**Affiliations:** ^1^Breast Center, Kameda Medical Center, 929 Higashi-cho, Kamogawa, Chiba 296-8602, Japan; ^2^Siemens Japan K.K. Healthcare Sector, 3-20-14 Higashi-Gotanda, Shinagawa-ku, Tokyo 141-8644, Japan

## Abstract

Proton MR spectroscopy of the mammary gland area is used to be considered in the realm of basic research, but as a result of the advances in MR techniques, it is now being performed in ordinary clinical practice. It is particularly noteworthy that useful clinical data are now being accumulated with 1.5T MR units, which are the standard units. We think that, at this point, it is very important to systematically review the techniques, clinical applications, and future prospects of proton MR spectroscopy. We have performed proton MR spectroscopy with a 1.5T MR unit in over 3000 cases at our hospital. In this paper, we will comment on the current status of proton MR spectroscopy of the breast, primarily in regard to differentiation between benign and malignant lesions and prediction of the efficacy of chemotherapy while describing the data obtained at our hospital.

## 1. Introduction

MRI of the breast has been applied clinically for more than 20 years, and in Western countries methods of using, it has been established in the form of guidelines [1, 2]. The core applications of MRI of the breast lie in detecting, diagnosing, and evaluating the efficacy of treatment of breast cancer [1, 2]. Today, however, besides the information on tumor morphology and blood flow obtained by contrast-enhanced MRI, it has become possible to obtain a variety of function images and molecular information, and PET and MR spectroscopies are representative studies that are capable of actually being used clinically.

Choline is considered an important metabolite in proton MR spectroscopy in the mammary gland area. Cholines are substances that have attracted interest in regard to every organ and disease, and because they are precursors of the phospholipids that compose cell membranes, increases in choline signals are thought to reflect increased membrane synthesis. In the mammary gland area, choline shows a promise of enabling differentiation between benign and malignant tumors and of serving as an indicator of tumor activity and viability. Clinically, the attention has been focused on differentiation between benign and malignant tumors and prediction of the efficacy of chemotherapy.

## 2. Metabolites Observed by Proton MR Spectroscopy of the Breast

The markers that are useful in breast diseases are centered at 3.2 ppm and are generally referred to as the choline peak. However, *myo*-inositol, taurine, and so forth are included in the choline peak in addition to such compounds as choline (Cho), phosphocholine (PC), and glycerophosphocholine (GPC), which are parts of the membrane lipid metabolic pathways, and because of the closeness of the ranges of their chemical shifts, they are observed as a single peak when measurements are made in vivo.

At the cellular level, GPC is higher than PC in normal breast tissue, and since a marked increase in PC and decrease in GPC have been demonstrated as a result of carcinogenesis [3], the main component in breast cancer is PC [3]. Moreover, it has been reported that if we examine minute chemical shifts even with a 1.5 T MR unit, it is possible to separate and observe a 3.22-3.23 ppm (PC) peak and a 3.27-3.28 ppm peak (GPC/taurine/*myo*-inositol), and that they are useful in differentiating between benign and malignant tumors [4].

Another peak observed besides choline is the lactose peak (3.8 ppm). The lactose peak is characteristic of the lactation period, and choline has also been found to be detected at 3.2 ppm during that period [5]. The choline observed in the lactating breast matches the choline (3.27 ppm) observed in benign diseases [4, 6].

## 3. Basic Summation: Imaging Techniques

Knowledge of imaging techniques is essential for proton MR spectroscopy of the breast. We will comment on each of the basic items in relation to imaging.

### 3.1. Voxel Size

In contrast to the central nervous system area, because of the presence of normal tissue, fat, and so forth, in the mammary glands, the magnetic fields in the voxels tend to be nonhomogeneous. Thus, when analyzing the spectra of tumors, it is important to set the voxels so that tissue outside the tumor is excluded. However, because there is a tradeoff between voxel size and acquisition time, a certain voxel size is unavoidable in order to obtain a high signal-to-noise ratio (SNR).

Assuming the addition of MR spectroscopy before or after approximately 20 to 30 minutes contrast-enhanced MRI, we think being able to perform MR spectroscopy in approximately 10 minutes would be ideal, and a minimum voxel size of about 15 mm × 15 mm × 15 mm would therefore appear to be appropriate for lesions with a diameter of 1 cm or greater.

### 3.2. Shimming

In contrast to the central nervous system, manual shimming as well as autoshimming is essential to make the magnetic fields of the voxels homogeneous. With manual shimming, we use full width at half-maximum (FWHM) or T2* for reference, and 20–30 Hz is the approximate target for FWHM.

### 3.3. Sequence

**Figure 1:**
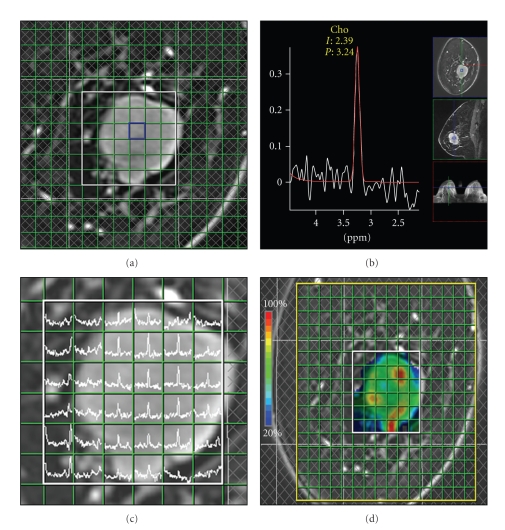
Chemical shift imaging (CSI) of breast cancer. ((a), (b)) Spectroscopy data acquire multitarget volume and multivoxels (a). TR = 1500 ms, TE = 270 ms, voxel size = 10 × 10 ×15 mm^3^, the time of acquisition = 7 minutes. The spectrum spans from 2.0 to 4.5 ppm (b). The choline (Cho) peak was detected at 3.24 ppm. ((c), (d)) Both spectral map (c) and metabolite map (d) are made by spectroscopy data. Choline peak is displayed in color map (d). High-signal intensity area (color: red) indicates high-signal intensity from breast cancer.

When the tumor is large, chemical shift imaging (CSI) is sometimes even used as a means of assessing the internal nonhomogeneity of the tumor (Figure 1). However, because multivoxelization is performed by using the point spread function by phase encoding, the same as for MRI, contamination from adjacent voxels occurs. Because of this defect, single-voxel spectroscopy is now the standard method. A spin-echo type, point-resolved spectroscopy sequence (PRESS) [7] is generally used in order to obtain single-voxel spectroscopy data.

When selecting echo time (TE) of proton MR spectroscopy, the decision as to whether to use a short TE or long TE involves making a tradeoff between signal intensity and signal contrast. A high SNR is obtained with short TE whereas long TE is superior in terms of ability to separate the contrast signal from the fat signal. With long TE (135–270 ms), the signal intensity decreases, but the fact that the fat signal also decreases means an improvement in ability to detect the choline signal [8].

### 3.4. Fat Suppression

Application of MR spectroscopy to the whole body has recently increased greatly, and many methods of fat suppression have been developed. Band-selective inversion with gradient dephasing (BASING) [9] is one of the latest fat suppression techniques and makes it possible to suppress any chemical shift spectrum signal desired by incorporating an additional pulse into the PRESS sequence. The pulses used for suppression, such as chemical-shift selective (CHESS) excitation pulses [10], are positioned in the form of prepulses immediately before the excitation pulses whereas with BASING the pulses for suppression are positioned within the body of the sequence itself. BASING enables suppression of the peak of any chemical shift desired, and it has been applied to peak editing, and so forth. This method is characterized by being little affected by T_1_ or B_1_.

### 3.5. Water Suppression

It is preferable for the water peak to persist to some extent in order to be able to use it as a standard to compute the chemical shift of a metabolite. While it is possible to perform water and fat suppression simultaneously by BASING, we use CHESS for water suppression at our hospital because of its strong suppressive effect. It is possible to set the intensity of the residual water peak by setting the waiting time after applying the CHESS pulse. We actually set the waiting time after applying the CHESS pulse at 200 ms.

### 3.6. Quantification

Two methods are available for quantification when performed by comparison with known concentrations: the internal reference method [11] and the external reference method [12]. In the internal reference method, the water signals in measurement voxels are used to quantify the target metabolites, whereas in the external reference method, a phantom is set up close to the measurement site, and the concentration in the volume in the phantom is used as the standard. Both methods have advantages and disadvantages. We use the external reference method to perform quantification at our hospital. 

With the internal reference method, the water content of the voxels changes during chemotherapy [13], and that is a potential pitfall with using it. That is why the external reference method was chosen at our hospital. The greatest disadvantage of the external reference method is the need to perform an additional independent measurement as an external reference, and it also requires correction for the partial volume effect and separate calibration experiments. Furthermore, since the T1 and T2 values of choline used were the values cited, there is the possibility of errors being introduced between these values and the actual values in the patients. In addition, because of patient movements, accurate correction is difficult even with frequency correction. Thus, the quantification method itself is beset with several problems.

As stated above, quantification is still challenging. However, quantification is essential in order to compare changes in the amount of choline for the patients treated with neoadjuvant chemotherapy. We believe that quantification is very useful and requires dedicated multidisciplinary team including radiologists, medical physicists, physicists, and chemists.

### 3.7. Imaging Method at Our Hospital

At our hospital, we perform proton MR spectroscopy with a 1.5 T unit (MAGNETOM Avanto; Siemens AG Healthcare Sector, Erlangen, Germany) and circularly polarized (CP) and four-channel phased-array breast coil (breast matrix coil; Siemens AG Healthcare Sector, Erlangen, Germany) [6]. The parameters used for MR spectroscopy are TR/TE = 1620/270; voxel size = 15 × 15 × 15 mm^3^; acquisitions = 256; spectral width = 1,000 Hz; data points = 1,024; and the time of acquisition is 7 minutes. Postcontrast coronal and sagittal T1-weighted MR images are used as scout images for voxel placement.

We have tried performing the following procedure for quantification by the external reference method.

A cylindrical bottle phantom (*syngo* GRACE external phantom; Siemens AG Healthcare Sector, Erlangen, Germany) 4.0 cm high and 2.5 cm in diameter was inserted behind the breast coil and fixed in position. The phantom was filled with 1.25 g of NiSO_4_6H_2_O per 1000 g of H_2_O. Proton MR spectroscopy of the phantom was performed immediately after the MR spectroscopy examination of the breast lesion. The scan was performed without water suppression. The voxel size was 7 × 7 × 15 mm^3^, and the acquisition time was 4 seconds.

The following formula was used to calculate the scaling factor:

(1) $$\begin{array}{cc} & \text{scaling}\;\text{factor}\\ & \;=\left(\frac{10^{6}}{{\text{MW}}_{\text{H}2\text{O}}}\right)\times \left(\frac{n_{\text{H}2\text{O}}}{n_{\text{Cho}}}\;\right)\times \left(\frac{f_{\text{T}1\text{H}2\text{O}}}{f_{\text{T}1\text{Cho}}}\right)\\ & \;\;\times \left(\frac{f_{\text{T}2\text{H}2\text{O}}}{f_{\text{T}2\text{Cho}}}\right)\times \left(\frac{{\text{voxel}}_{\text{H}2\text{O}}}{{\text{voxel}}_{\text{Cho}}}\right)\times \left(\frac{{\text{coilsens}}_{\text{H}2\text{O}}}{{\text{coilsens}}_{\text{Cho}}}\right),\\ f_{\text{T}1} & =1-\exp\;\;\left(-\frac{\text{TR}}{\text{T}1}\right),\;f_{\text{T}2}=\exp\;\;\left(-\frac{\text{TE}}{\text{T}2}\right), \end{array}$$

where *n*_Cho_ and *n*_H2O_ are the numbers of  ^1^H nuclei of choline molecules and water molecules, respectively. The scaling factor can be converted to molar concentration by correcting for the number of ^1^H nuclei per molecule and the molecular weight of the solvent (MW_H2O_). The *f*_T1_ and *f*_T2_ relaxation factors were corrected by using the equation for relaxation times.

The relaxation times (T1 and T2) of the phantom water were measured. T1 was calculated from the images obtained with two different TRs according to the spin-echo sequence, with TE maintained constant, and T2 was calculated from the images obtained with 16 different TEs with TR maintained constant. The mean T1 was 375 ms, and the mean T2 was 270 ms. The T1 and T2 values in the report published by Baik et al. (T1_Cho_ = 1513 ms, T2_Cho_ = 269 ms) [14] were adopted as the T1 and T2 values for choline in vivo. The coil sensitivities indicate the signal intensity of the external reference phantom and the signal intensity within the imaging area. The signal intensity within the imaging area was obtained on proton-weighted images (TE/TR, 15/5000) with a solution phantom introduced into the measurement breast coil that was the same as the external reference solution.

### 3.8. Data Processing and Spectral Interpretation at Our Hospital

The spectroscopic data processing protocol was saved and linked to the measurement protocol within the *syngo* software (Siemens AG Healthcare Sector, Erlangen, Germany) to ensure that data processing was identical for each measurement. The spectra were processed by zero-filling the 1028 data points to 2048 data points by applying a Gaussian apodization function of 1.5 Hz before fast Fourier transformation. The scaling factor was 12096. We fitted the choline peak and water peak with a Gaussian function that ranged from 3.18 to 3.32 ppm for choline and was 4.7 ppm for water. The peak line widths were restricted according to the following settings; the Cho peaks were recognized as Cho when the FWHM was less than 10 Hz; when the FWHM was 10 Hz or more, the peaks no longer fit.

Phase correction was performed manually. The residual water signal was used for reference (4.7 ppm), and the frequency of any resonance detected in the 3.00–3.50 ppm spectral region was recorded. Choline peaks at 3.21–3.23 ppm are assigned to phosphocholine (PC) and were evaluated by using a threshold SNR of 2 [15, 16]. When positive for choline, water subtraction and baseline correction with a sixth-order polynomial fit were applied to obtain the flat baseline of an MR spectrum. The normalized choline signal, which was calculated automatically, was recorded. When negative for choline, the numerical value of choline was recorded as zero. 

## 4. Clinical Summation: Usefulness for Differential Diagnosis

Thus far, reports on clinical research on proton MR spectroscopy of the breast have centered on differentiating between benign and malignant lesions. However, the possibility of using 1.5 T to detect biomarkers of breast cancer has also been reported in recent years. Below we comment on the possibilities related to the diagnostic power of proton MR spectroscopy for breast lesions. 

### 4.1. Differentiation between Benign and Malignant

The major papers on the diagnostic performance of proton MR spectroscopy as a means of differentiating between benign and malignant tumors that were published between 1998 and 2009 are listed in Table 1 [5, 15–23]. According to the data in the nine other papers after excluding our own [23], sensitivity ranged from 70% to 100%, and specificity ranged from 67% to 100%. Sensitivity in the nine articles as a whole was 88% (165/187), and specificity was 88% (126/144), and the diagnostic performance reported was related to lesions of 1 cm or greater in size.

The special characteristic of our own study [23] is that we performed proton MR spectroscopy before biopsy in order to exclude any effects of bleeding or inflammation after a biopsy, and Breast Imaging Recording and Data System (BI-RADS-) MRI [24] category 4 and 5 lesions were the subject of our study. There were 171 cases, more lesions than in any of the other studies, but a comparison with the previous studies showed that sensitivity was very low (44%; 40/91). We think the reasons for the low sensitivity were that the target lesions were small and that nonmass lesions were included. Actually, when restricted to masses measuring 15 mm or more, sensitivity improved to 82% (28/34). 

The first study of nonmass lesions by proton MR spectroscopy was reported by Bartella et al. [25], and the results were favorable, that is, a sensitivity of 100% and a specificity of 85%. By contrast, the results for sensitivity in our own research [23] were very low, 32% (9/28), and specificity was 75% (12/16) (Table 2). One of the reasons for this is thought to be differences between the types of cancers that were the subject of the studies. Ductal carcinoma in situ (DCIS) accounted for 17% (2/12) of the cancers in the report by Bartella et al. [25] whereas they accounted for 89% (25/28) of the cancers in our study. The morphologic patterns of DCIS were variable on MRI and included a branching-ductal pattern and a scattered clumped pattern in cases with low tumor density at histology and confluent clustered rings in those with high tumor density at histology [23]. The proportion of DCIS might affect the sensitivity of MR spectroscopy for nonmass lesions. Therefore, the diagnostic performance for nonmass lesions of proton MR spectroscopy with the 1.5 T MR unit currently being used does not appear to be satisfactory, and we think it will be necessary to assess stronger magnetic fields in the future. It also appears that in the future, it will be necessary to improve the coils, optimize the measurement sequence, and increase the precision of the data postprocessing.

However, false-negative cases have been reported even in relatively large invasive cancers, and in addition to invasive ductal carcinoma, they have been reported in medullary carcinoma [19, 21], mucinous carcinoma [23], and apocrine carcinoma [23]. The existence of these false-negative cases corroborates the fact that biopsy must not be avoided on the basis of negative proton MR spectroscopy findings alone. Fibroadenoma [5, 15, 16, 19, 22, 23], tubular adenoma [17, 18], intraductal papilloma [23], mastopathy (including benign proliferative disease) [5, 18, 22, 23], inflammatory lesions with atypia [16], and atypical ductal hyperplasia [16, 22] have been reported.

### 4.2. Choline Elevation in Breast Parenchyma

The elevated choline level in breast cancer may be associated with increased membrane synthesis by replicating cells, however, benign tissues, such as proliferative fibroadenoma, may also produce a positive-choline signal [26].

**Figure 2:**
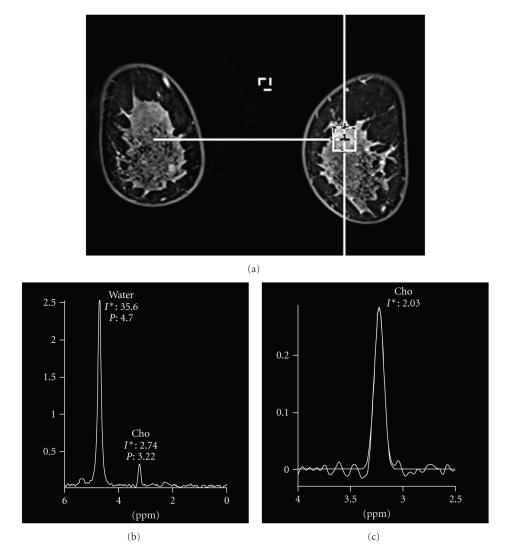
Proton MR spectroscopy of the normal breast parenchyma. Coronal 3D contrast-enhanced fat-suppressed MR image (TR/TE, 5.2/2.3) shows no suspicious findings (a). Single-voxel spectrum shows a choline (Cho) peak at 3.22 ppm (b). Water subtraction and baseline correction are applied (c). The normalized choline (Cho) signal is 2.03.

When we measured the proton MR spectroscopy of the breast parenchyma in 920 cases at our hospital, however, we observed a choline peak in 12% (113/920), and 32% of the choline peaks (36/113) were consistent with the choline that is seen in malignancy (PC; 3.21–3.23 ppm) (Figure 2). The exact reason for this phenomenon is unknown, but the increase in choline in the breast parenchyma may be greater than previously thought. This is important in terms of clinical diagnosis, and caution is required because of the possibility of intermingling by very weak choline in the surrounding area, especially when a voxel that is larger than the lesion is placed.

### 4.3. Effect of the Contrast Medium

Since breast lesions are usually detected by MRI after contrast medium has been injected, we tried performing proton MR spectroscopy after contrast-enhanced MRI, and the gadolinium contrast medium appeared to have a very slight effect the MR spectrum of the lesion. Lenkinski et al. [27] used on a 3 T MR unit to conduct a study of the effect of six gadolinium contrast media (Magnevist, MultiHance, Omniscan, Optimark, ProHance, and Dotarem) on MR spectroscopy performed on phantoms and a rat model of breast cancer. The results obtained with the phantom showed that the width of the choline peak broadened with three of the contrast media, that is, Magnevist, MultiHance, and Dotarem, and the volume of the peak decreased to an average of about 40%. The use of negatively-charged chelates may be associated with the underestimation of the choline level present in the lesion, and their conclusion was to recommend the use of neutral chelates for MR spectroscopy of the breast.

**Figure 3:**
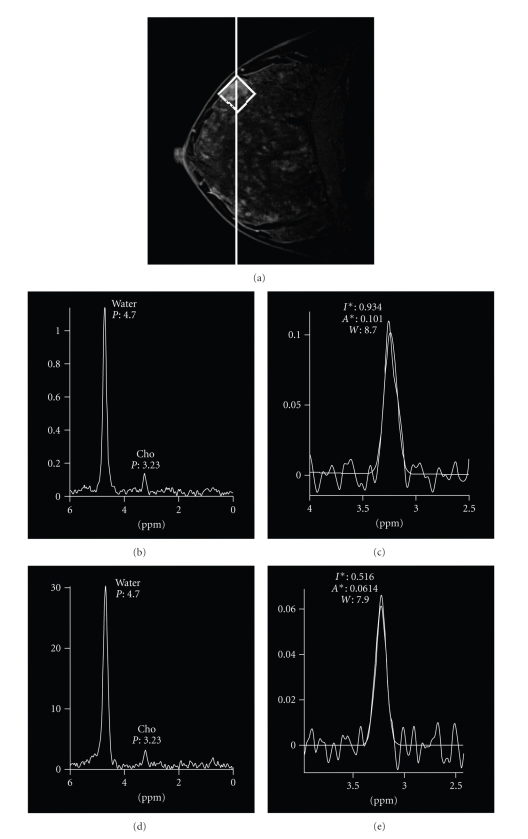
The effect on the choline spectrum by using two different contrast media (negatively-charged chelates and neutral chelates). Sagittal 3D contrast-enhanced fat-suppressed MR image (TR/TE, 4.0/2.2) shows an enhanced mass (fibroadenoma) (a). Single-voxel spectrum using gadopentetate dimeglumine (Magnevist) ((b), (c)). Single-voxel spectrum using gadodiamide (Omniscan) ((d), (e)).

We attempted to determine the effect on the choline spectrum at our hospital by using two different contrast media (negatively-charged chelates and neutral chelates), Magnevist (gadopentetate dimeglumine, Gd-DTPA) and Omniscan (gadodiamide, Gd-DTPA-BMA). We used each of the two contrast media on a different day in 30 patients who had been found to have an enhanced mass, and we compared the changes in width and height of the choline peaks. The Wilcoxon signed-rank test was used to statistically analyze the data. Approval to conduct the study was obtained from the ethics committee. The results did not show any statistically significant changes in the width or height of the choline peak due to the contrast media (Figure 3), and no statistically significant differences in FWHM or T2* during shimming were observed in the same cases. We think these findings suggested that the impact of the contrast medium in clinical settings in which 1.5 T MRI is used is very mild. MR spectroscopy is more strongly impacted by the location of the tumor (skin and vicinity of the chest wall) and the imaging conditions, and the impact of the contrast medium appears to be trivial by comparison.

### 4.4. Possibilities as a Means of Biomarkers

The potential of proton MR spectroscopy as a means of biomarkers of breast cancer has already been investigated in many ex vivo studies. Lean et al. [28] reported being able to differentiate Grade I and II breast cancers from Grade III breast cancers based on MR spectroscopy findings in an ex vivo study of fine-needle aspiration specimens. We have reported similar findings in vivo, as described below [29]. In our study, the choline levels measured by proton MR spectroscopy with a 1.5-T unit correlated well with the histological prognostic factors, that is, nuclear grade, estrogen receptor status, and triple-negative lesion status. It is hoped that in vivo research on proton MR spectroscopy to detect biomarkers will contribute to breast cancer therapy, including drug therapy.

## 5. Clinical Summation: Application to Neoadjuvant Chemotherapy

The role of diagnostic imaging in neoadjuvant chemotherapy lies in diagnosing the spread of residual tumors after chemotherapy and in judging the efficacy of the chemotherapy [2]. There are two very important points in regard to the diagnosis of the spread of residual lesions after chemotherapy: “the pattern of tumor spread before chemotherapy” and “the pattern of tumor shrinkage after chemotherapy, [30]” and when this information is taken into consideration, it appears possible to perform breast-conserving surgery safely. Judging the efficacy of chemotherapy, on the other hand, means evaluating the chemosensitivity of the tumor in vivo. Many reports have shown that the variety of information provided by MRI (tumor diameter, changes in volume, and changes in blood flow) is more useful than the information provided by palpation or ultrasonography [31–34]. A great deal of hope is now being placed in MR spectroscopy and PET molecular images. 

### 5.1. Prediction of Therapeutic Efficacy

As stated above, since proton MR spectroscopy has a role in the evaluation of treatment of breast diseases by providing molecular information, it is regarded as an effective method for monitoring the activity of breast cancer and for early prediction of the efficacy of chemotherapy [35–43]. Accurate assessment of the response to neoadjuvant chemotherapy is one of the critical factors for optimizing the chemotherapy regimen and planning further surgery. The greatest advantages of early prediction of efficacy are being able to reduce unnecessary adverse effects, prevent treatment delays, and avoid unnecessary administration of expensive drugs that do not show promise of being effective. We describe several of the problems below.

### 5.2. Voxel Size: Variable or Fixed

Since methods that alter MR spectroscopy voxels according to the size of the tumor during the course of breast cancer chemotherapy are routine [36, 40], quantification is essential in order to compare changes in the amount of choline. However, the reproducibility of the quantitative values themselves is a problem, because when the voxels are altered, shimming accuracy and the SNR change. We therefore gave priority to obtaining stabilized voxels that could be compared before and after treatment and tried a method in which we fix voxel size and position the voxel at the same site as the lesion. 

However, because this is not the standard method, we first investigated its accuracy by making comparisons with PET/CT in the same patients [39]. The results of the comparisons of the amount of choline (integral values) obtained by MR spectroscopy and the standardized uptake value (SUV) obtained by PET/CT showed that the changes in the two perfectly paralleled each other [39]. 

When we fixed the voxel size, there appeared to be major advantages from a clinical standpoint in terms of being able to fix the scanning parameters, such as receiver gain, and easily observe increases in choline within the unit voxel. On the other hand, one of the disadvantages of this technique is that tumor metabolism can only be observed partially in tumors larger than the voxel size. The other is that the tissue surrounding the tumor is included in the voxel when the tumor becomes smaller than the voxel as a result of regression. However, if MR spectroscopy is used to identify nonresponders early, this disadvantage is avoidable. Further studies are needed to examine the advantages and disadvantages of this technique in a larger number of patients.

### 5.3. PET/CT versus Proton MR Spectroscopy

There have been more reports of using PET/CT for early prediction of the efficacy of chemotherapy for breast cancer than of using proton MR spectroscopy. The study on the largest number of cases (*n* = 64) reported that the evaluations after 2 courses of therapy were more accurate than the evaluations after 1 course or after 3 courses [44]. We conducted a study to determine whether proton MR spectroscopy or PET/CT is more suitable for early prediction of efficacy [41], and we conducted it after two courses of chemotherapy when prediction of efficacy by PET/CT also was reported to be the most accurate. The results of the study by the external reference method with a fixed voxel size (15 × 15 × 15 mm) in 16 cases showed that proton MR spectroscopy and PET/CT were equivalent [41]. However, whether MR spectroscopy is as good as PET has not been firmly established. Further studies are needed.

### 5.4. Problems in Early Prediction of Efficacy

Neoadjuvant chemotherapy usually consists of an anthracycline-based regimen followed by taxane-based regimens, but because the early evaluations by MR spectroscopy are no more than evaluations of the initial anthracycline-based regimen and are not always consistent with the pathological evaluation after the completion of chemotherapy, evaluations of efficacy performed during the initial anthracycline-based regimen alone are inadequate for early prediction of the efficacy of this method. Although the number of cases has been small, there have been cases in which the anthracycline-based regimen was ineffective, but the efficacy of subsequent taxane-based regimens was excellent, and cases in which the opposite was true. We therefore assessed early prediction of efficacy by MR spectroscopy in which treatment with an anthracycline-based regimen alone was performed [43], and the results indicated that the changes in choline after the second cycle of chemotherapy as determined by quantitative MR spectroscopy may be a more sensitive means of predicting the pathological response than changes in tumor size are. We think that it is preferable to try making early predictions of the efficacy of neoadjuvant chemotherapy for each of the individual drugs.

### 5.5. Appropriate Timing of Early Predictions of Efficacy

Based on the results described above, proton MR spectroscopy may not be inferior to PET/CT as a diagnostic method for early prediction of efficacy in breast cancer. In the next step, it will be necessary to determine whether it is possible to predict efficacy by proton MR spectroscopy earlier than after 2 courses. In a study on 14 cases, Meisamy et al. [36] found that the changes in the amount of choline detected at 4T within the first 24 hours after a single course correlated with clinical efficacy after the completion of chemotherapy. We showed that the results of an evaluation by proton MR spectroscopy at 1.5 T after one course (several days before the start of the second course) correlated with pathological size after the completion of chemotherapy [42]. However, the same as in the study by PET/CT [44], it was also possible to underestimate the efficacy of treatment after one course more than after two courses. Proton MR spectroscopy is a technique that is still in the development stage in the breast disease. In the future, it will be necessary to carefully evaluate the proper timing of early prediction of the efficacy of chemotherapy because the underestimated data could be an obstacle to clinical application.

## 6. Conclusion

Proton MR spectroscopy in breast diseases provides molecular information that is useful clinically. The very basis of the spectroscopy method relies on the use of magnets with higher field strength to separate the diagnostic resonances. With the growing availability of 3 T systems, the use of breast MR spectroscopy for diagnosing lesions and monitoring response to neoadjuvant chemotherapy will become routine clinical practice. However, as stated above, there have not been very many reports on differential diagnosis between benign and malignant lesions or on early prediction of the efficacy of neoadjuvant chemotherapy. The reason is that many problems must be resolved, including the optimal measurement sequence for proton MR spectroscopy, differences between MR units, spectrum interpretation, postprocessing adjustments, and methods of choline quantification (internal reference method or external reference method). However, if these problems are not resolved, it will be impossible to ensure the safety and reliability of being able to use it for cancer treatment (drug therapy). Standardization of proton MR spectroscopy and multicenter collaborative research appear to be essential.

## Figures and Tables

**Table 1:** The diagnostic power of proton MR spectroscopy for breast lesions.

Study	year	No. of malignant lesions	No. of benign lesions	Sensitivity (%)	Specificity (%)	No. of false-positive findings	PPV (%)
Roebuck et al. [17]	1998	10	7	70	86	1	88
Kvistad et al. [5]	1999	11	11	82	82	2	82
Cecil et al. [18]	2001	23	15	83	87	2	90
Yeung et al. [19]	2001	24	6	92	83	1	97
Jagannathan et al. [20]	2001	32	14	81	86	2	93
Tes et al. [21]	2003	19	27	89	100	0	100
Huang et al. [15]	2004	18	12	100	67	4	82
Bartella et al. [16]	2006	31	26	100	88	3	91
Sardanelli et al. [22]	2009	19	26	84	88	3	84
Tozaki et al. [23]	2009	91	80	44	85	12	77
Tozaki et al. [23]*	2009	34	16	82	69	5	85

*masses measuring 15 mm or more.

**Table 2:** The diagnostic power of proton MR spectroscopy for nonmass lesions.

Study	year	No. of malignant lesions	No. of benign lesions	Sensitivity (%)	Specificity (%)	No. of false-positive findings	PPV (%)
Bartella et al. [25]	2007	12	20	100	85	3	80
		invasive ductal carcinoma; 10 ductal carcinoma in situ; 2				fibroadenoma; 1 inflammatory lesions with atypia; 1 atypical ductal hyperplasia; 1	
Tozaki et al. [23]*	2009	28	16	32	75	4	69
		invasive ductal carcinoma; 2 microinvasion; 1 ductal carcinoma in situ; 25				benign proliferative disease; 4	

*nonmass lesions.
